# CNX-011-67, a novel GPR40 agonist, enhances glucose responsiveness, insulin secretion and islet insulin content in n-STZ rats and in islets from type 2 diabetic patients

**DOI:** 10.1186/2050-6511-15-19

**Published:** 2014-03-25

**Authors:** Venkategowda Sunil, Mahesh Kumar Verma, Anup M Oommen, Manojkumar Sadasivuni, Jaideep Singh, Dasarahalli N Vijayraghav, Bhawna Chandravanshi, Jayalaxmi Shetty, Sanghamitra Biswas, Anilkumar Dandu, Yoganand Moolemath, Marikunte V Venkataranganna, Baggavalli P Somesh, Madanahalli R Jagannath

**Affiliations:** 1 Connexios Life Sciences Pvt Ltd, #49, “SHILPA VIDYA” 1st Main, 3rd Phase, JP Nagar, Bangalore 560078, India

**Keywords:** β-cells, CNX-011-67, Glucose stimulated insulin secretion, GPR40, Neonatal streptozotocin, Type 2 Diabetes mellitus, Islet insulin content

## Abstract

**Background:**

GPR40 is a G-protein coupled receptor regulating free fatty acid induced and also glucose induced insulin secretion. We generated neonatally-streptozotocin-treated female rats (n-STZ) and treated them with CNX-011-67, a GPR40 agonist to examine the role of GPR40 in modulation of glucose metabolism, insulin secretion and content.

**Methods:**

Female n-STZ animals were orally administered with CNX-011-67 (15 mg/kg body weight, twice daily) or with vehicle for 8 weeks (n = 8 per group). Glucose tolerance in treated animals and insulin secretion, islet insulin content and gene expression in isolated islets were determined. Islets from type 2 diabetic mellitus (T2DM) patients were treated with different concentrations of glucose in presence or absence of CNX-011-67 and insulin secretion was measured.

**Results:**

Treatment of n-STZ rats with GPR40 agonist CNX-011-67 enhanced insulin secretion in response to oral glucose load on day 0 and this response persisted during the treatment period. The treatment also produced a ‘memory effect’ during which insulin secretion in response to oral glucose load remained enhanced, for a week, even in absence of the agonist. Activation of GPR40 enhanced responsiveness of islets to glucose and increased glucose induced insulin secretion and islet insulin content. An increase in islet mRNA expression of GCK, PDX1, insulin and PC was also observed. Acute treatment of islets from n-STZ rats with GPR40 agonist enhanced cellular ATP content. Activation of GPR40 enhanced mitochondrial calcium level in NIT-1 insulinoma cells. CNX-011-67 increased insulin secretion in islets from T2DM patients which were non-responsive to increased glucose concentration

**Conclusions:**

Our data provide evidence that activation of GPR40 with CNX-011-67 stimulates glucose metabolism, enhances glucose responsiveness, increases insulin secretion and content and that pharmacological activation of GPR40 will prove beneficial for treatment of T2DM.

## Background

Type 2 Diabetes Mellitus (T2DM) is growing in epidemic proportions both in the developed and developing countries and is estimated to affect 438 million humans world-wide by the year 2030 (World Diabetes Foundation). Normally the β-cell responds to an increment in glucose with an increase in insulin to maintain normoglycemia [1,2]. In response to oral glucose an ‘early phase’ insulin secretion is observed within the first 30 min of ingestion which is reduced in subjects with impaired glucose tolerance suggesting that an impaired first phase is perhaps the earliest detectable abnormality in individuals destined to develop T2DM [3,4]. With onset of β-cells failure there is first a steady increase in postprandial and fasting glucose concentrations leading finally to the development of overt diabetes [1,5-7].

Several therapeutic approaches have been adopted to prevent and/or delay the defects in β-cell function [8,9]. Of relevance to this study is the role of the G protein coupled receptor 40 (GPR40), also known as Free Fatty Acid Receptor 1 in the regulation of β-cell function. GPR40 is highly expressed in human β-cells, brain and endocrine cells of the gastrointestinal tract and is activated by medium and long chain saturated and unsaturated fatty acids and enhance insulin secretion by activating cytosolic calcium flux [10-12]. Over-expression of GPR40 increases whereas its knockout decreases insulin secretion [13,14] indicating its important role for insulin secretion and glucose tolerance. Similarly, its expression is reduced in T2DM islets and is positively correlated with insulin secretion [15]. Hence, GPR40 is an important therapeutic target to treat T2DM. In fact a number of small molecule agonists have been shown to modulate glucose stimulated insulin secretion. Different GPR40 agonists such as GW9508, TAK-875, AS2575959, AMG837 and phenyl propanoic acid derivatives have shown increased insulin secretion in both insulinoma cells and/or in animal models [16-20]. Among these agonists, TAK-875 has been shown to prevent β-cells dysfunction [21] in animal model of diabetes and improves glycemic control in T2DM patients [22,23]. However, none of these abovementioned molecules have been reported to increase insulin secretion in n-STZ model which has high β-cell stress.

While the precise primary defects that trigger β-cell defects are not fully understood, islets from patients with T2DM have been reported to display reduced glucose oxidation [24,25], reduced ATP content in response to an acute glucose stimulation [26] and reduced glucose stimulated insulin secretion (GSIS) [24,25,27]. These changes are associated with a decrease in expression of glucokinase (GCK), glucose transporter 1 and 2 (GLUT1 and 2), and alterations in genes involved in insulin granule exocytosis [24,28,29].

β-cell population in diabetics is reduced by 30% when accompanied by deposition of islet amyloid [30,31] and a 50% decline in β-cell volume is observed in patients with impaired fasting glucose [32]. The similar pathology was reported in neonatal streptozotocin (n-STZ) rats [33-36]. Also stress in β-cell of this model is very high which is similar to the stress seen in T2DM patient’s islets.

We have reported previously that chronic treatment of male ZDF rats (insulin resistant model with elevated blood glucose and free fatty acid levels) with CNX-011-67 increased insulin secretion, decreased blood glucose and reduced β-cells apoptosis without affecting body weight [37]. We have shown that chronic β-cell stress mediated by glucolipotoxic conditions reduces GSIS, islet insulin content, ATP levels and expression of genes such as GCK, PDX1 and insulin [38]. Chronic treatment of CNX-011-67 was able to overcome these defects in β-cells functions [37]. In the present study we were interested to evaluate chronic activation of GPR40 in the neonatal streptozotocin (n-STZ) rats which manifests reduced β-cell number, decreased GSIS and severe β-cell stress as seen in T2DM patient islets [33-36]. Chronic treatment with CNX-011-67 resulted in improved glucose responsiveness, phasic glucose stimulated insulin secretion and increased islet insulin content. CNX-011-67 also increased glucose stimulated insulin secretion in human islets from T2DM patient. These results suggest that activation of GPR40 by CNX-011-67 will provide a novel therapeutic approach to improve long-term glycemic control in T2DM patients by regulating β-cell biology.

## Methods

### Reagents

Glucose was estimated using Accu-check glucometer (Roche Diagnostics, Germany). Ultra-sensitive insulin ELISA kit (Crystal Chem Inc, USA) was used to determine plasma insulin levels. Insulin was estimated in *in-vitro* studies using “ultra-sensitive insulin assay kit” (Mercodia, Sweden) according to the manufacturer’s instructions.

### Animals

In-bred Wistar rats were provided commercial pelleted food (Provimi, India) and water *ad libitum* and maintained on a 12 h light and 12 h dark cycle during the studies. All animal experiment procedures were approved by the Institutional Animal Ethics Committee of Connexios Life Sciences and were in accordance with the guidelines of Committee For The Purpose Of Control And Supervision On Experiments On Animals (CPCSEA) Govt. of India.

### Oral glucose tolerance test (OGTT)

After a 16 h fast, CNX-011-67 (5 mg/kg or 15 mg/kg body weight) or vehicle was administered 30 min or 45 min prior to administration of glucose (2 g/kg b.wt) by oral gavage (n = 8 per group). Blood samples were collected from the tail vein 30 min or 45 min before treatment and at 0, 10, 20, 30, 60 and 120 min after glucose load for estimating glucose and insulin.

### Generation and treatment of n-STZ rats

Following breeding, pups were administered 50 mg/kg, i.p of STZ (Sigma-Aldrich, St. Louis, USA) or vehicle on day 2 and 3 of birth and allowed to be with their mothers until weaning at 21 days of age. Subsequently, only animals that were confirmed as glucose intolerant in an oral glucose tolerance test (at the age of 17 weeks) were included in the study. More than 95% of the animals that were administered with STZ were glucose intolerant at 17 week and the rest of the animals developed glucose intolerance 2 weeks later.

Three experimental groups were studied, namely, sham control, n-STZ control and n-STZ + CNX-011-67 (15 mg/kg b.wt.). CNX-011-67 was dissolved in water and administered twice daily by oral gavage (15 mg/kg b.wt.). The n-STZ control animals were administered vehicle (water). Four protocols were followed:

**Protocol 1**: n-STZ animals were treated with CNX-011-67 (twice daily, 15 mg/kg body weight) or vehicle for 12 weeks (n = 8 per group) and oral glucose tolerance test was performed on day 0 and week 8.

**Protocol 2**: n-STZ animals were treated with CNX-011-67 (twice daily, 15 mg/kg body weight) or vehicle for 8 weeks (n = 8 per group) and islet mRNA was isolated for RT-PCR based analysis of gene expression.

**Protocol 3**: n-STZ animals were treated with CNX-011-67 (twice daily, 15 mg/kg body weight) or vehicle for 8 weeks (n = 8 per group) and treatment was withdrawn for 1-week (ninth week of the study). OGTT was performed after week 8 and again after one week of drug withdrawal (9th week). Agonist treatment was reintroduced and continued for a further 3 weeks after which glucose stimulated insulin secretion and insulin content were determined in islets isolated from the animals.

**Protocol 4**: Islets isolated from n-STZ rats were acutely treated with CNX-011-67 and ATP content was measured.

Daily food intake and body weight were recorded for all the animals in the above protocols

### Islet isolation, gluose stimulated insulin secretion and islet insulin content measurement

Animals were killed under anesthesia (due to ethical considerations; all groups of animals were similarly treated) and pancreata were digested with collagenase-II (Sigma-Aldrich, St. Louis, USA) followed by islet separation in a density gradient centrifugation using Histopaque (Sigma diagnostics, St. Louis, MO, USA) as described earlier [39]. Size-matched islets were handpicked under a stereomicroscope (Nikon) and used for GSIS assay. Briefly, islets were seeded in 24-well culture plates in 1 ml of Krebs-Ringer bicarbonate HEPES (KRBH) buffer with 2.8 mM glucose and incubated at 37°C for 1 h. Islets were then treated with 1 μM of CNX-011-67 in the presence of low (5.6 mM) or high (16.7 mM) glucose for 2 h. The culture supernatant was collected and stored at −70°C for measurement of insulin. Islets were then lysed in lysis buffer and islet insulin content was measured. Both secreted insulin and islet insulin content were normalized with total protein measured using Bradford’s reagent (Bio-Rad). Amount of secreted insulin was also normalized with islet insulin content and represented as % of islet insulin content.

Human islets from cadevar T2DM donars were procured from Prodo lab, USA. Size matched islets were hand picked and incubated in KRBH containing 2.8 mM glucose for 1 h. Islets were then incubated with different concentrations of glucose with or without CNX-011-67 (1 μM) for 2 h. The supernatent was collected and used for insulin measurement. Four islets were used in each replicate for the GSIS.

### Measurement of mitochondrial and cytosolic calcium flux in N1T1 cells

NIT1 cells (ATCC) were cultured in Ham’s F12 medium (Sigma-Aldrich) supplemented with 15% FBS, 2 mM L-glutamine, 100 U/ml penicillin and 100 μg/ml of streptomycin (All reagents from GIBCO, USA). For experiments, NIT-1 cells were seeded at a density of 40,000 cells/well in 96-well plates and incubated for 72 h. Cells were loaded with Fluo-3 AM (Invitrogen, 2.5 μM) dye for cytosolic calcium measurement or Rhod-2 AM dye (Invitrogen, 4 μM) for mitochondrial calcium measurement for 60 min and washed twice with HEPES buffered saline solution (HBSS) to remove unbound dye. Following a basal measurement, cells were treated with low glucose (2.8 mM), high glucose (16.7 mM) or high glucose with 1 μM of CNX-011-67 and Ca^2+^ levels were measured for 4 min at 6-s intervals using a Biotek Synergy 2 fluorimeter with resulting flux being represented as arbitrary fluorescence units (AFU) [40]. Calcium levels represented refer to net response with background fluorescence corrections. There were no oscillations observed and the response was sustained for nearly 12 minutes.

### Quantitative Real-time PCR (qPCR)

Total RNA was extracted from islets using Tri-reagent (Sigma, St. Louis, MO, USA), followed by chloroform extraction and isopropyl alcohol precipitation. This total RNA (500 ng) was used for cDNA synthesis by reverse transcription (ABI, Foster City, CA, USA). mRNA levels were quantified using SYBR Green PCR Master Mix (Eurogenetic, Belgium) using 5 ng of cDNA. Genes analyzed in the present study were GCK, PC, PDX1 and insulin. For quantification of gene expression, β-actin was used as internal control (primer sequences are available upon request).

### Measurement of β-cell ATP and inositol-tri-phosphate (IP3) content

After preincubation at 2.8 mM glucose for 60 min, groups of ten islets were incubated in tubes containing 0.5 ml KRBH buffer supplemented with 2.8 or 11 mM glucose with or without 300 nM CNX-011-67 for 60 min for ATP and 5 min for IP3 measurement. The islets were then washed twice with 1X cold PBS and lysed in lysing buffer provided by the manufacturer. ATP and IP3 concentrations were measured using ATP determination kit (Molecular Probes, USA) or Rat IP3 ELISA Kit (CUSABIO biotech, China) in a Multi-Mode Micro plate Reader (SpectraMax M5e, Molecular Devices, USA).

### Histology and immunohistochemistry of β-cells

Pancreata were dissected out from the animals and fixed in 10% buffered neutral formalin for 48 h. The entire pancreas was collected, weighed, processed and paraffin-embedded. Each block was serially sectioned (4 μm) throughout its length to avoid any bias from regional changes in islet distribution and islet cell composition, and sections were mounted on clean glass slides. Three serial sections were obtained every 100 microns. One was used for hematoxylin and eosin staining, and the remaining for immunofluorescence staining for insulin and PDX1.

Immunofluorescence staining for insulin and PDX1 was carried out using a goat anti-mouse FITC conjugated and goat anti-rabbit Alexafluor conjugated secondary antibody direct labeling technique, respectively. Sections were incubated for 1 h with anti-insulin (Abexome, India) and anti-PDX1 (Abcam, UK) antibodies. Thereafter, FITC- and Alexafluor-conjugated secondary antibodies were applied for 30 min. After staining, sections were mounted in fluromount (Sigma). All hematoxylin and eosin sections were taken for histological evaluation under light microscope.

Images were captured using Zeiss fluorescence microscope using progres® capture pro 2.1 camera. Quantitative evaluation of total β-cell mass was performed using a computer-assisted image analysis (progres®capturepro) software. The relative volume of β-cells was determined by a stereological morphometric method, calculating the ratio between the area occupied by immunoreactive cells and that occupied by total pancreatic cells. Total β-cell mass per pancreas was derived by multiplying this ratio by the total pancreatic weight. The total β-cell nuclei from all stained section were counted, to derive number of β-cells per islet.

### Electron microscopy studies

Pancreatic tissue was fixed in 3% gluteraldehyde for 24 h, washed with phosphate buffer twice and post-fixed with 1% Osmium tetroxide. Tissues were then washed with phosphate buffer twice and processed in different grades of alcohol for dehydration and propylene oxide for clearing. Tissues were infiltrated with a mixture of propylene oxide: araldite (1:1) and incubated on a rotator overnight at room temperature. Tissues were transferred to fresh and pure araldite, incubated and then embedded. Blocks were allowed to polymerize for 48 h at 60°C. Ultra thin sections were stained with uranyl acetate and lead citrate and scanned in a Transmission Electron Microscope.

### Statistical Analysis

Data values are expressed as mean ± S.E.M. Significance of differences among groups was determined using One-way analysis of variance followed by Dunnett’s post-test or by Students unpaired t-test. P value summary: (*) <0.05, (**) <0.01 and (***) <0.001 respectively, when compared with vehicle control.

## Results

### Phenotypic characterization of n-STZ rats prior to the start of treatment

Table 1 gives body weight, fasting glucose and insulin levels in the neonatally STZ treated adult (n-STZ) rats and sham control rats at week 17 of age, 1 week before commencement of treatment with CNX-011-67. There was no significant difference in body weight and fasting glucose between the sham control and n-STZ groups but the n-STZ animals displayed decreased insulin area under curve (AUC) and severe glucose intolerance during ‘Oral Glucose Tolerance Test’ (OGTT). Fasting insulin levels in n-STZ animals had declined to 0.174 ± 0.04 ng/ml as compared to 0.31 ± 0.05 ng/ml recorded in the sham control animals.

**Table 1:** Phenotypic characterization of n-STZ rats (prior to treatment commencement)

	**Body weight (g)**	**Fasting glucose (mg/dl)**	**Fasting insulin (ng/ml)**	Glucose AUC (OGTT) (mg/dl)*min	Insulin AUC (GSIS) (ng/ml)*min
Sham (n = 32)	202 ± 6	83 ± 3	0.31 ± 0.05	14791 ± 255	98 ± 8.6
n-STZ (n = 55)	203 ± 3	80 ± 1.7	0.17 ± 0.04*	31617 ± 931***	79 ± 5.14

Statistical comparison between sham and n-STZ group was conducted by Students t-test (Two-way, unpaired: *p < 0.05 and ***p < 0.001).

### Effect of GPR40 agonist treatment on insulin secretion and glucose levels with and without glucose load in Wistar rats

**Figure 1:**
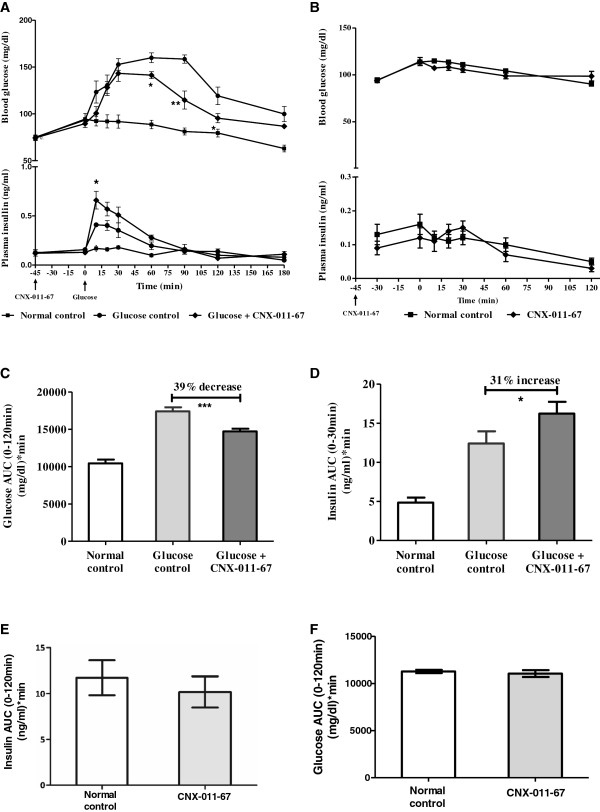
**Effect of CNX**-**011-67 in normal wistar rats and rat islets- OGTT glucose, insulin and GSIS.** Effects of CNX-011-67 on blood glucose and insulin levels in normal Wistar rats with **(A)** and without glucose load **(B)**. The rats were dosed with vehicle or CNX-011-67 by oral gavage at −45 min, blood samples obtained at the time intervals indicated in the graphs for glucose and insulin estimation. Effects of CNX-011-67 on total blood glucose **(C)** & insulin **(D)** AUC during the OGTT in Wistar rats. Effects of CNX-011-67 on total insulin **(E)** & blood glucose **(F)** AUC during the OGTT without glucose load in Wistar rats. The percent inhibition of glucose levels was calculated based on the glucose AUC during the 0-120 min OGTT for each group after subtracting the values from those of the normal (no drug and no glucose) control group. Data in all panels are mean ± SEM (n = 8/group). Statistical comparison between control and treatment group was conducted by one-way ANOVA followed by Dunnett’s post test correction. (*P < 0.05 and ** P < 0.01).

In normal Wistar rats, oral administration of 2 g/kg glucose resulted in a significant increase in insulin secretion (0–30 min AUC 12.45 ± 1.55 vs 4.85 ± 0.63) when compared to control animals while treatment with CNX-011-67 (5 mg/kg body weight) significantly enhanced insulin secretion (0–30” AUC 16.23 ± 2.05 vs 12.45 ± 1.55, 31% increase, P < 0.05) in response to oral glucose (Figure 1A lower panel and D) when compared with glucose control. There was a significant decrease in glucose AUC in CNX-011-67 treated animals when compared to untreated animals (0–120 min AUC 14716 ± 360 vs 17400 ± 535, 39% decrease, P < 0.001) (Figure 1A upper panel and C). In absence of oral glucose challenge, treatment with CNX-011-67 (15 mg/kg body weight) did not either increase insulin secretion (0–30 min AUC 10.18 ± 1.7 vs 11.73 ± 1.9) (Figure 1B lower panel and E) or decrease blood glucose levels (0–120 min AUC 11049 ± 362 vs 11272 ± 176) (Figure 1B upper panel and F).

### Effect of GPR40 agonist treatment on early phase insulin secretion and glucose tolerance in n-STZ rats

**Figure 2:**
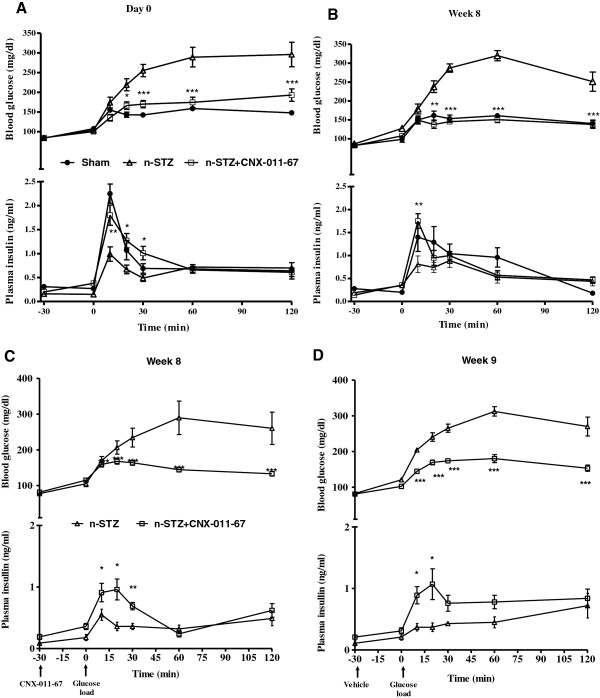
**Effect of CNX-011-67 on OGTT glucose and insulin levels in n-STZ rats.** On day 1 **(A)** and week 8 **(B)**. Blood samples were collected from the tail vein at the intervals indicated in the graph for estimating insulin and glucose levels. The lower panel represents the OGTT glucose and insulin levels at the end of the study in n-STZ-CNX-011-67 rats on week 8 **(C)** and week 9 **(D)** which is one week after agonist withdrawal. In all the experiments, animals were fasted for 16 h and oral glucose administered at 2 g/kg load. On week 8, CNX-011-67 was administered 30 min before oral glucose load. Blood samples were collected from the tail vein at the intervals indicated in the graph for estimating insulin and glucose levels. Open triangle: n-STZ; Closed circle: Sham control; Open square: n-STZ-CNX-011-67. Data in all panels are mean ± SEM (n = 8/group). Statistical comparison between control and treatment group was conducted by one-way ANOVA followed by Dunnett’s post test correction. (*P < 0.05, ** P < 0.01 and *** P < 0.001).

The n-STZ group of animals showed loss of insulin secretion capacity in response to an oral glucose challenge (Figure 2A, lower panel). Insulin secretion in response to oral glucose load increased (1.8 fold) in the n-STZ-CNX-011-67 treated animals after administration of the first dose itself (day 0) leading to a significant improvement in glucose tolerance when compared to the (untreated) n-STZ animals (AUCglucose 20057 ± 885 vs 30433 ± 865 respectively, n = 8; P < 0.01; Figure 2A upper panel). The improvement in glucose tolerance was almost similar to the glucose tolerance observed in the sham control animals (AUCglucose 17900 ± 217). Chronic administration of CNX-011-67 to n-STZ animals showed no impact on body weight and feed consumption. Histological analysis did not show any organ related toxicity (data not shown). After 8 weeks of treatment with CNX-011-67 agonist, a similar increase in insulin secretion (2-fold, Figure 2B lower panel) in response to oral glucose load and improvement in glucose tolerance was observed in the n-STZ-CNX-011-67 animals compared to the untreated n-STZ animals (17225 ± 786 vs 32480 ± 1697 respectively, n = 8; P < 0.01; Figure 2B upper panel) and this improved glucose tolerance was again similar to the glucose tolerance observed in the sham control animals group (18094 ± 501). Similarly, the increase in insulin secretion in first 30 min, on day 0, led to a significant improvement in glucose tolerance in the n-STZ-CNX-011-67 animals when compared to the untreated n-STZ animals (area under the 0–30 min glucose curve: 4232 ± 182 vs 5725 ± 358, respectively, n = 8; P < 0.01; Figure 2A upper panel) and was similar to the glucose tolerance observed in the vehicle treated sham control animals (AUCglucose 4221 ± 102). A similar increase in insulin secretion and improvement in glucose tolerance at 30 min post oral glucose load was also observed after 8 weeks of treatment with the n-STZ-CNX-011-67 agonist. A significant aspect of the n-STZ-CNX-011-67 agonist action is the steep increase in the early phase insulin secretion (~1.8–2 fold increase) at 15 min and the significant glucose clearance within 30 min of the oral glucose load. These data indicate that treatment of n-STZ animals with the CNX-011-67 enhanced glucose sensitivity of β-cells and almost normalized insulin secretion in response to oral glucose load.

### Effect of withdrawal of CNX-011-67 treatment on insulin secretion in response to oral glucose load

To understand if the chronic effects induced by CNX-011-67 agonist were maintained in the long term n-STZ-CNX-011-67 animals were administered the agonist for 8 weeks and then the treatment was withdrawn for a period of 1 week. Oral glucose tolerance test was performed at end of 8 weeks and again after one week of agonist withdrawal. Since the half life of CNX-011-67 agonist is 6 h in Wistar rats (data not shown), it is unlikely that the compound administered a week earlier remained in circulation in the n-STZ-CNX-011-67 animals. The similar profiles of the oral glucose tolerance tests performed prior to (increase in 27% insulin AUC in n-STZ-CNX-011-67 in 8th week, P < 0.001, Figure 2C lower panel) and 1 week after agonist withdrawal (35% increase in insulin AUC in the 9th week, P < 0.05, Figure 2D lower panel) respectively indicates that the β-cells in the n-STZ-CNX-011-67 animals remained responsive to glucose even in the absence of the agonist. It appears that the CNX-011-67 induced β-cell population in the n-STZ-CNX-011-67 remains functional and sensitive to stimulatory glucose concentrations even in absence of the agonist.

### Effect of CNX-011-67 on GSIS, insulin content of islets, β-cell mass and number

**Figure 3:**
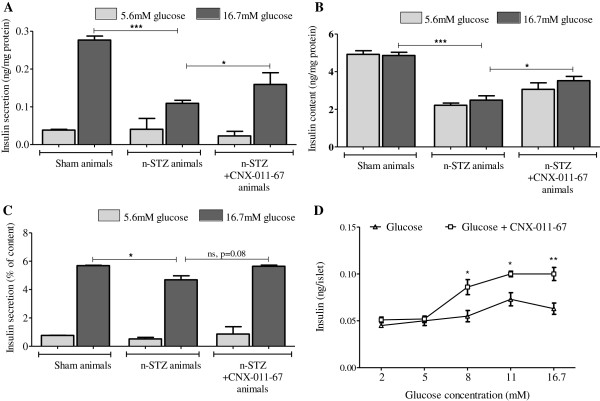
**CNX-011-67 increases islet insulin secretion and content.** Insulin secretion **(A)**, islet insulin content **(B)**, ratio of secreted insulin to insulin content represented as percentage **(C)** of islets from sham control, n-STZ and n-STZ-CNX-011-67 animals after treatment with CNX-011-67 for 8 weeks. Freshly isolated rat islets were preincubated with 2.8 mM glucose for 30 min and later incubated with 5.6 mM and 16.7 mM glucose for 120 min. Both insulin secretion and islet insulin content were measured. Secreted insulin and islet insulin content are presented as ng of insulin/mg protein. **(D)** CNX-011-67 increases insulin secretion in human islets from T2DM patients. The islets from T2DM did not show glucose responsiveness as elevated glucose concentration (8 mM) could not increase insulin secretion. CNX-011-67 treatment resulted in increased glucose responsiveness as CNX-011-67 facilitated insulin secretion only at higher glucose concentration. Values represent mean ± S.E.M (*n* = 4). Statistical analysis was performed by Students t-test (Two-way, unpaired: *P < 0.05, **P < 0.01 and ***P < 0.001).

Freshly isolated size-matched islets from sham control, n-STZ and n-STZ-CNX-011-67 animals (8 weeks treatment) were tested *in vitro* in a static glucose stimulated insulin secretion (GSIS) assay. Islets from sham control animals displayed the expected steep increase in insulin secretion upon high glucose stimulation while islets from the n-STZ animals, in sharp contrast, displayed a weak insulin secretory response (60% decrease compared to sham control, P < 0.001) indicating a significant loss of β-cell sensitivity to glucose (Figure 3A). However, islets from the n-STZ-CNX-011-67 animals displayed a 55% increase (P < 0.05) in insulin secretory (Figure 3A) response that was significantly greater than that observed in islets from n-STZ animals indicating a robust improvement in β-cell sensitivity to glucose upon CNX-011-67 treatment. Analyses of islet insulin content from the three groups showed a similar trend (Figure 3B). Insulin content in the islets from n-STZ animals was reduced (48% decrease compared to sham control, P < 0.001) indicating a potential reduction in insulin biosynthesis. In contrast, islets from n-STZ-CNX-011-67 animals exhibited a 41% increase (P < 0.05) in insulin content compared to the n-STZ group and more importantly, an increase in insulin biosynthesis was observed upon exposure to 16.7 mM glucose (Figure 3B) indicating increased response to glucose stimulation. When expressed as percent of islet insulin content, insulin secretion from islets prepared from n-STZ animals displayed a 22% decrease (P < 0.05, Figure 3C) while islets from n-STZ-CNX-011-67 animals displayed only a 4% decrease when compared with sham controls. However, the islets from n-STZ-CNX-011-67 animals displayed a 20% increase, though non-significant (P < 0.08), when compared with islets from n-STZ animals (Figure 3C).

Since pancreatic islets from n-STZ animals resemble T2DM human islets in terms of increased stress and reduced insulin secretion, we sought to measure insulin secretion in human islets obtained from T2DM cadaver donors and studied impacts on CNX-011-67 treatment. As shown in Figure 3D, T2DM human islets did not secrete elevated levels of insulin in response to physiological stimulatory glucose concentration (8 mM). Insulin secretion was increased to some extent only at 11 mM glucose concentration and further increase in glucose level showed no further insulin secretion. Treatment of T2DM human islets with CNX-011-67 increased insulin secretion only at stimulatory glucose concentration (8 mM) and had no impact at lower glucose levels (upto 5 mM) (Figure 3D). These data thus further indicate that activation of GPR40 by CNX-011-67 can increase insulin secretion in a glucose dependent manner.

**Figure 4:**
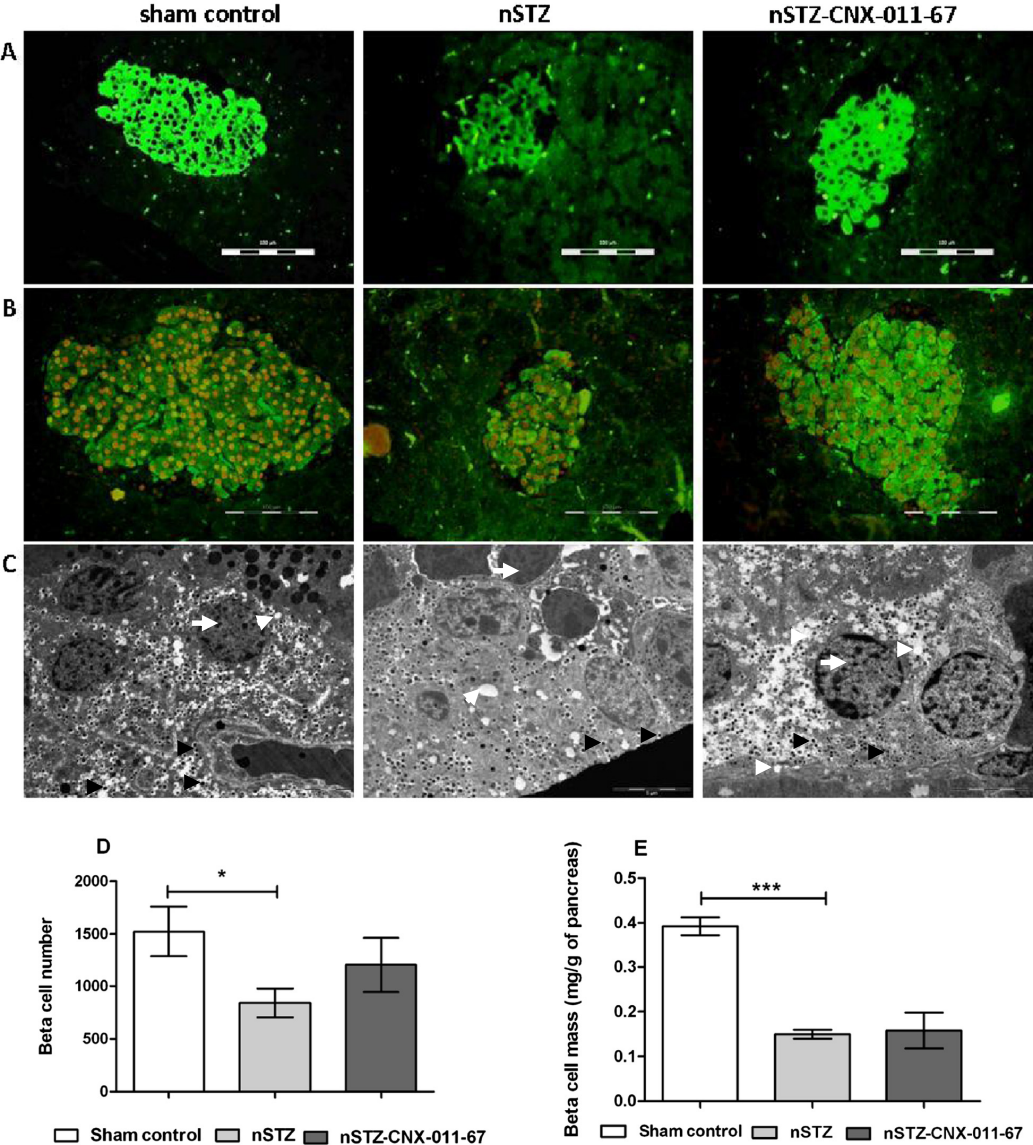
**CNX-011-67 improves islet insulin content, β-cell mass and number-immuno fluorescence staining and elctron microscope studies of CNX-011-67 treatment in islets and β-cell.** Sections from sham control, n-STZ and n-STZ-CNX-011-67 Islets stained for insulin content using Immuno fluorescent staining (FITC; green) **(A)**. Double immuno fluorescence staining for PDX1 (red; alexafluor 555) and insulin (green; FITC) representing all groups **(B)**. Electron microscope images of β-cells from sham control, n-STZ and n-STZ-CNX-011-67 animals, treated animals showed improvement and increased number of dense-core insulin granules when compared with n-STZ animals. Mitochondria and endoplasmic reticulum health have restored in treatment **(C)**. White arrow: nucleus, white arrowheads: mitochondria, black arrowheads: insulin vesicles. The n-STZ animals showed decreased β-cell number and mass when compared with normal control animals. The treated animals exhibited non significant increase in the β-cell number and β-cell mass **(D & E)**. Images were taken at 40× magnification for immunohistochemical analysis and 4800× magnification for electron micrographs. Scale bar: 5 μm. Data in all panels are mean ± S.E.M (n = 6). Statistical analysis was performed by Students t-test (two-way, unpaired: *P < 0.05 and ***P < 0.001).

Immunohistochemical analysis of islet sections from sham control, n-STZ and n-STZ-CNX-011-67 animals revealed a modest increase in insulin (Figure 4A) and PDX-1 (Figure 4B) immunofluorescence while electron microscopic studies indicated presence of higher number of electron-dense insulin granules in n-STZ-CNX-011-67 animals (Figure 4C). However, the effect of treatment with CNX-011-67 on β-cell number and β-cell mass was statistically non-significant (Figure 4D & E). Hematoxylin and eosin staining of pancreas showed no detectable change in morphology or structure (data not shown).

### Effect of CNX-011-67 on mitochondrial and cytosolic calcium content

**Figure 5:**
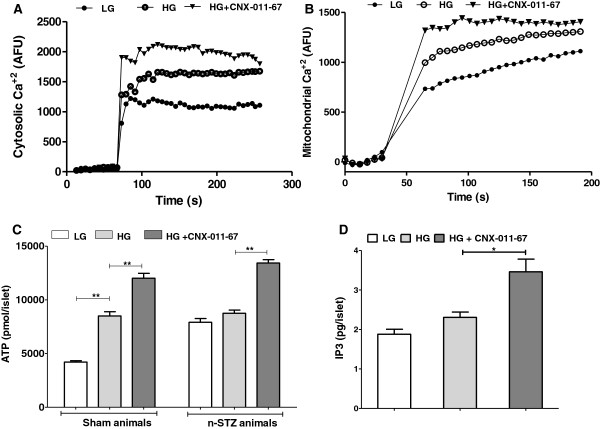
**CNX-011-67 increases both cytosolic and mitochondrial calcium in NIT1 cells and ATP levels in rat islets.** Cultured NIT1 cells were loaded with Fluo-3 AM dye for cytosolic calcium measurement **(A)** and Rhod-2 AM dye for mitochondrial calcium measurement **(B)** for 60 min. Following a basal measurement, cells were treated with low glucose (2.8 mM), high glucose (16.7 mM) or high glucose (16.7 mM) with 1 μM of CNX-011-67 followed by measurement of Ca^2+^ levels for 4 min at 6-s intervals. The resulting flux was represented as arbitrary fluorescent units (n = 3). **(C)** Islet ATP content from sham control and n-STZ animals. **(D)** Islet IP3 content in normal Wistar rat islets. Islets were preincubated with 2.8 mM glucose for 1 h and later incubated with low glucose (LG, 2.8 mM), high glucose (HG, 11 mM) or high glucose (11 mM) with 1 μM of CNX-011-67 for 60 min for ATP and 5 min for IP3 measurement. ATP (pmol) and IP3 (pg) content are presented per islet. Values represent mean ± S.E.M (n = 4). Statistical analysis was performed by Students t-test (Two-way, unpaired: * P < 0.05 and ** P < 0.01).

As GPR40 is coupled to Gα-q/11 protein and specific ligands like oleic acid induce an increase in cytosolic calcium, we explored whether CNX-011-67 increased cytosolic Ca^2+^ levels. While exposure to stimulatory glucose concentration in NIT-1 cells led to a 43% increase in cytosolic [Ca^2+^] consistent with earlier reports [41-44], the presence of CNX-011-67 resulted in a further increase by 24% in cytosolic [Ca^2+^] (Figure 5A). GPR40 agonism by CNX-011-67 thus led to a significant augmentation of cytosolic [Ca^2+^] over that induced by stimulatory glucose concentrations. CNX-011-67-mediated increase in cytosolic [Ca^2+^] levels were however dependent on presence of calcium in extracellular medium as inhibition of voltage dependent calcium channels by nitrendipine abrogated increase in cytosolic [Ca^2+^] levels (data not shown). As GPR40 activation enhanced endoplasmic reticulum Ca^2+^ release, we next examined if this could lead to an increase in mitochondrial [Ca^2+^]. In NIT-1 cells, while exposure to stimulatory glucose concentrations led to a 20% increase in mitochondrial [Ca^2+^], presence of CNX-011-67 caused a further increase by 13% in mitochondrial Ca^2+^ levels (Figure 5B).

### Effect of treatment with CNX-011-67 on islet ATP and inositol-tri-phosphate (IP3) content in n-STZ islets

Freshly isolated islets from sham control animals displayed enhanced ATP synthesis when exposed to stimulatory glucose concentration and this was enhanced further in the presence of CNX-011-67 (P < 0.01, Figure 5C). On the contrary, ATP synthesis in islets freshly isolated from n-STZ animals was already high, as previously reported [45], even at low glucose concentrations when compared to sham control, and exposure to stimulatory glucose levels induced only a marginal increase in ATP synthesis as expected. However, the presence of CNX-011-67 restored ATP synthesis in the islets from n-STZ animals in response to stimulatory glucose concentrations to almost control levels suggesting enhanced glucose metabolism (P < 0.01, Figure 5C). IP3 content in islets was also significantly enhanced by addition of CNX-011-67 in agreement with earlier observations [46] that the signal transduction of GPR40 involves phospholipase C activation, and elevation of intracellular IP3 leading to intracellular calcium release (Figure 5D).

### Treatment with CNX-011-67 enhances expression of factors regulating glucose metabolism and insulin synthesis

**Figure 6:**
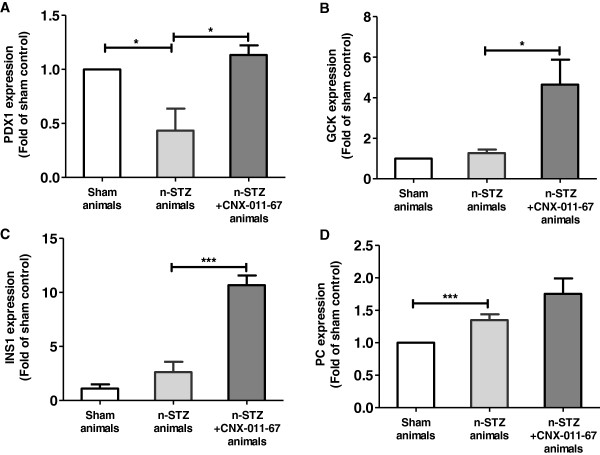
**Effect of CNX-011-67 on islet gene expression.** mRNA levels of PDX1 **(A)**, GCK **(B)**, INS1 **(C)** and PC **(D)** in islets from sham control, n-STZ and n-STZ-CNX-011-67 rats were measured as mentined in the methods. The mRNA levels of all the genes were expressed relative to β-actin mRNA. Values represent mean ± S.E.M (n = 4). Statistical analysis was performed by Students t-test (Two-way, unpaired: * P < 0.05 and *** P < 0.001).

To investigate the molecular mechanisms underlying CNX-011-67 mediated increase in insulin secretion and insulin content, we examined the expression of factors regulating glucose metabolism and insulin synthesis in islets isolated from sham control, n-STZ and n-STZ-CNX-011-67 rats (Figure 6). In n-STZ animals while expression of PDX-1 was decreased (P < 0.05, Figure 6A), expression of pyruvate carboxylase (PC, Figure 6D) was significantly increased and that of glucokinase (GCK, Figure 6B) and insulin genes (Figure 6C) remained unchanged when compared with sham controls. Expression of all these four factors showed a significant increase in the islets from n-STZ-CNX-011-67 rats indicating that chronic treatment with CNX-011-67 has a beneficial effect on expression of genes that are critical for regulating glucose metabolism and insulin synthesis.

## Discussion

The objective of this study was to investigate the therapeutic effects of CNX-011-67, a specific GPR40 agonist, in ameliorating the defects in glucose responsiveness of β-cells and insulin secretion in the neonatally STZ-treated adult Wistar rat model. Female rats were selected for the present study as loss in insulin secretion capacity in neonatally STZ treated adult animals remain uniform irrespective of the sex of the animals [47]. The n-STZ model represents a model having reduced β-cell number and increased β-cells stress indicating thus the β-cells pathology in this model is similar to what seen in human diabetic patients [30-32]. Acute treatment with CNX-011-67 induced a significant increase in insulin secretion in response to oral glucose stimulation and chronic treatment for eight weeks induced significant changes in expression of genes involved in glucose metabolism and insulin synthesis leading to enhanced insulin content in islets.

A number of small molecules such as GW9508, TAK-875, AS2575959 and AMG837 have been synthesized to activate GPR40 to induce insulin secretion [16-20] in different model systems. However, none of these molecules have shown for their potency to increase insulin secretion in n-STZ animals, a model where β-cell stress is high. Moreover, it is not clear from present literature whether GPR40 activation has any impact on β-cells glucose metabolism which is an important factor for insulin release. Though, Alquier et al. [14] reported that GPR40 knock out did not alter fuel metabolism under normal condition, the same can not be corroborated for GPR40 activation under disease conditions. In this report and in the earlier study [37] we provide evidences, though indirectly, that CNX-011-67 increases glucose metabolism in β-cells.

Since, increased insulin secretion for prolonged period can cause β-cells exhaustion which can instead complicate the pathology, it become important to study whether the increase in insulin secretion by GPR40 activation has any impact on islet insulin content. Interestingly, we have observed that CNX-011-67 can actually increase the islet insulin content despite increasing insulin secretion thereby indicating that insulin synthesis is improved. This was further substantiated by increased expression of insulin and PDX1.

It is well known that increase in β-cell glucose metabolism and ATP synthesis activates voltage-gated Ca^2+^ channels culminating in influx of extracellular Ca^2+^ and insulin granule exocytosis [48,49]. Acute treatment with CNX-011-67 significantly enhanced cytoplasmic and mitochondrial [Ca^2+^] in NIT-1 cells and increased IP3 and ATP levels in islets prepared from normal Wistar rats and n-STZ rats, respectively. These observations suggest that acute exposure of n-STZ islets to CNX-011-67 perhaps leads to an increase in islet cytoplasmic and mitochondrial [Ca^2+^] levels [50,51] leading to activation of the dehydrogenases of the citric acid cycle and enhanced ATP content observed in the n-STZ islets (Figure 5C). The defective Calcium-activated oxidative metabolism and ATP synthesis reported in islets in n-STZ animal models [52] is likely overcome by acute treatment with CNX-011-67 and results in enhanced insulin secretion in response to stimulatory glucose concentrations.

The above sequence of molecular alterations perhaps explains the enhanced insulin secretion observed in the n-STZ-CNX-011-67 rats on day 0 itself, following the first exposure to CNX-011-67 (Figure 2A). The acute phase of insulin release observed in the first 10 min following oral glucose stimulation in the n-STZ-CNX-011-67 rats on day 0 is attributable to mechanisms such as enhanced mitochondrial [Ca^2+^], glucose metabolism and ATP synthesis, that together trigger exocytosis of a small number of insulin granules from the readily releasable pool in a K_ATP_ channel-dependent manner as described previously [53-55]. Such an effect is also evident in the acute reversal of loss of insulin secretion observed in islets from n-STZ rats after exposure to CNX-011-67 suggesting that the mechanism of action of the agonist ensures glucose responsiveness and enhances hormone secretion. This explains, in part, the acute increase in insulin secretion observed on day 0 of treatment with CNX-011-67. A similar acute effect on glucose stimulated insulin secretion was also evident in normal Wistar rats (Figure 1). The robust insulin secretion observed after eight weeks of treatment with the GPR40 agonist suggested an improvement in β-cell number or function. The enhanced glucose stimulated insulin secretion and insulin content observed in cultured islets prepared from the n-STZ-CNX-011-67 rats when exposed to 16.7 mM glucose (Figure 3A & B) indicates an improvement in glucose responsiveness of the β-cells and is also one reason for the sustained insulin secretion observed in the treated animals.

In the n-STZ animals, the reduced insulin secretion and insulin content can be explained by defective mitochondrial activity leading to reduced ATP synthesis in spite of normal expression levels of mRNA of GCK, insulin and PC genes involved in upstream events of glucose metabolism. The increase in mRNA levels of PDX1, GCK and PC in the n-STZ-CNX-011-67 rats indicates improved glucose metabolism (Figure 6 A, B & D) initiated by the agonist while the increase in insulin mRNA and higher ATP availability support higher insulin synthesis (Figures 6C and 5C).

Even though we have not provided any direct evidence to support that GPR40 activation enhances β-cell glucose oxidation our study provides indirect evidence to link activation of GPR40 with enhanced glucose metabolism in β-cells. Since glucose metabolism and ATP content are directly related in pancreatic β-cells and we observed an increase in ATP content hence we speculate that glucose metabolism is increased. Moreover, GCK expression is reported to be reduced after STZ treatment [56] leading to a reduced glucose metabolism, and with CNX-011-67 treatment we observed an up-regulated expression of GCK further indicating that glucose metabolism in enhanced after CNX-011-67 treatment.

The near-normal insulin secretory response to oral glucose maintained in n-STZ-CNX-011-67 rats even after the withdrawal of the agonist can be explained by the change in expression of multiple genes involved in glucose metabolism and insulin synthesis. For example, it has been previously reported that in Zucker diabetic fatty rats a reduced expression of GLUT2 was associated with reduced insulin response to higher glucose concentrations, and transgenic overexpression of GLUT2 rescued the mice from early death [57,58]. Treatment of n-STZ rats with sodium tungstate enhanced phosphorylation of PDX-1 and increased β-cell replication and insulin producing cells [25].

In the present study, the significant increase in islet insulin content and modest increase in area of insulin positive cells observed after 12 weeks treatment with CNX-011-67 most likely occurs due to a synergy resulting from a coordinated increase in expression of PDX1, and genes (insulin, GCK, and PC) involved in glucose metabolism and ATP synthesis. The electron micrographs not only indicate the preponderance of dense insulin granules in islets from n-STZ-CNX-011-67 rats, almost similar to the islets from sham control rats, but also show that there is an increase in the number of granules docked at the membrane and ready for release upon glucose stimulation. It may be mentioned here that treatment of Wistar rats with CNX-011-67 at 5 and 15 mg/kg did not increase serum GLP1 levels and hence the effects observed in the β-cells in this study are attributable to the direct activation of GPR40 on β-cells by CNX-011-67. We recently reported a similar increase in ATP synthesis, islet insulin content and enhanced early phase insulin secretion in male ZDF rats upon treatment with CNX-011-67 [37].

The decrease in insulin secretion in T2DM patients is attributed both to a loss in β-cell mass and to an increase in number of β-cells that respond poorly to glucose [59]. Pancreatic β-cells from diabetic patients display a decrease in expression of GCK, GLUT 1 and 2, genes involved in insulin granule exocytosis, show reduced glucose oxidation and ATP content resulting in reduced glucose stimulated insulin release. Further, patients with T2DM display a reduced early phase insulin secretion in response to oral glucose stimulation. The incretin glucagon like peptide-1 (GLP-1) has demonstrated trophic effects on β-cells as evidenced by increased differentiation of pancreatic ductal cells, reduced apoptosis and enhanced proliferation of β-cells in animal models [60] and incretin-based therapies have been developed to treat T2DM in humans. On account of its ability to enhance Gαq/11 signaling, GPR40 agonism represents a novel therapeutic approach to ameliorate the defects in insulin secretion observed in patients with T2DM. The phenotype of impaired glucose metabolism, lower content of insulin, lower rate of insulin biosynthesis and content and a persistent impairment of insulin release especially at higher glucose concentration appear to be similar in islets from adult n-STZ rats and from T2DM patients.

Chronic treatment of CNX-011-67 did not show any impact on body weight, feed consumption and pancreata histology and also no toxic effects were observed. Thus, CNX-011-67 is a novel, non-toxic, potent small molecule having insulin secretion ability in a glucose dependent manner. These results are in consistent with our earlier study [37] where we have shown insulin secreting ability of CNX-011-67 in male ZDF animal model without any detectable impact on feed intake or body weight. Taken together, CNX-011-67 is an orally available small molecule capable of enhanced insulin secretion in a glucose dependent manner. Therefore, the CNX-011-67 holds promise as a future therapeutic agent to meet the demand of insulin secretion in T2DM patients whose β-cells are under chronic stress.

## Conclusions

This study suggests that β-cells that are non-responsive to glucose, for various reasons, can be pharmacologically revived by CNX-011-67- mediated activation of GPR40 to synthesize and secrete insulin in response to glucose stimulation. The ability of CNX-011-67 to overcome the ‘glucose incompetence’ of islet β-cells and increase insulin secretion and content in response to stimulatory glucose concentrations has clear implications for treating T2DM.

## Abbreviations

GPR40: Gprotein coupled receptor 40; T2DM: Type 2 diabetes mellitus; n-STZ: Neonatelly straptozotocin treated; GSIS: Glucose stimulates insulin secretion; GCK: Glucokinase; GLUT: Glucose transporter; PC: Pyruvate carbozylase; IP3: Inositol-tri-phosphate; AUC: Area under curve; OGTT: Oral glucose tolerance test.

## Competing interests

The authors declare that they have no competing interests.

## Authors’ contributions

VS, JS, DNV, BC, JS, SB carried out the experiments; MKV, MKS, YM, AD designed the study and carried out the analysis. MKV, MOA, MVV and BPS interpreted the data and drafted the manuscript. MRJ supervised the progress and critically revised the manuscript. All authors read and approved the final manuscript.

## Pre-publication history

The pre-publication history for this paper can be accessed here:

http://www.biomedcentral.com/2050-6511/15/19/prepub
